# Bacterial secretion system functions: evidence of interactions and downstream implications

**DOI:** 10.1099/mic.0.001326

**Published:** 2023-04-21

**Authors:** Silindile Maphosa, Lucy N. Moleleki, Thabiso E. Motaung

**Affiliations:** ^1^​ Division of Microbiology, Department of Biochemistry, Genetics, and Microbiology, University of Pretoria, Hatfield, Pretoria, South Africa; ^2^​ Department of Plant and Soil Sciences, University of Pretoria, Hatfield, Pretoria, South Africa; ^3^​ Forestry and Agricultural Biotechnology Institute, University of Pretoria, Hatfield, Pretoria, South Africa

**Keywords:** Secretion systems, intersecretion system crosstalk, nutrient acquisition, horizontal gene transfer, bacteria-host interaction

## Abstract

Unprecedented insights into the biology and functions of bacteria have been and continue to be gained through studying bacterial secretion systems in isolation. This method, however, results in our understanding of the systems being primarily based on the idea that they operate independently, ignoring the subtleties of downstream interconnections. Gram-negative bacteria are naturally able to adapt to and navigate their frequently varied and dynamic surroundings, mostly because of the covert connections between secretion systems. Therefore, to comprehend some of the linked downstream repercussions for organisms that follow this discourse, it is vital to have mechanistic insights into how the intersecretion system functions in bacterial rivalry, virulence, and survival, among other things. To that purpose, this paper discusses a few key instances of molecular antagonistic and interdependent relationships between bacterial secretion systems and their produced functional products.

## Introduction

Systems of molecular secretion in bacteria promote pathogenicity and disease in diverse animal, human, and plant hosts. Depending on the lifestyle of the bacteria, secretion systems assume numerous key roles commonly entailing the transport of small molecules, nucleic acids, and proteins [1]. A suit of these secretory pathways dictates many aspects of bacterial biology that often maximize the success of bacteria and how pathogens impose adverse consequences on public, livestock, and plant health. For these reasons, knowledge behind the molecular mechanisms of these pathways has great implications for effective antivirulence drug discovery and subsequent management of bacterial infections.

Secretion systems are important for the biology of bacteria as transporters of proteins from the cytoplasm to the outer membrane and transporters of proteins from a donor cell to the environment or a recipient cell [1]. The latter type of transporters, which have evolved over time as nanomachines facilitating competition for resources and space, is the subject of a large number of excellent reviews that address, at large, how they facilitate bacterial competition and interaction with the environment, other bacteria, and hosts [1–3].

Secretion systems have mostly been analysed in Gram-negative bacteria (GNB) and, to some extent, Gram-positive bacteria (GPB) [4–8]. They are often designated as TXSSs, where X stands for any number from 1 to 11, including outer membrane vesicles (OMVs), sometimes called a type zero secretion system (T0SS) [2, 6, 9, 10]. A major unifying thread among pathogenic bacterial secretory pathways, not related to type, is how they primarily assemble into channels for the transport of proteins including different virulence factors. Upon release, these proteins travel into and through biological barriers to interact with host components and specific immune factors, and subsequently promote the reprogramming of several important cellular processes, ultimately leading to disease.

**Fig. 1.**
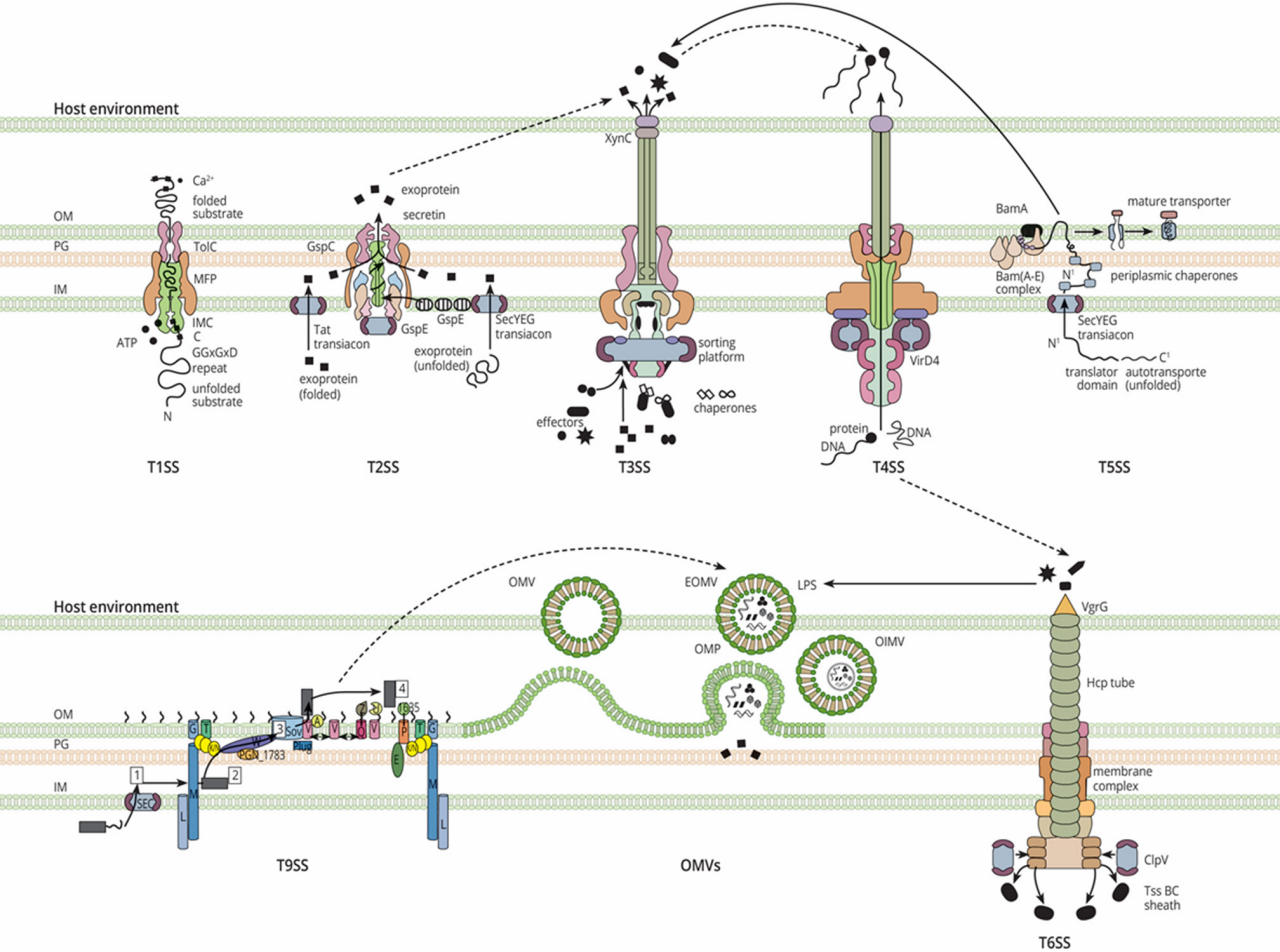
Secretion systems (type 1–type 6 secretion system) of Gram-negative bacteria and available evidence of crosstalk with other secretion systems. The figure shows the six secretion systems of GNB (T1SS-T6SS) and the dialogs they are involved in with other secretion systems. The dotted lines indicate interaction at both substrate and system levels, while solid lines indicate interaction at either substrate or system level.

In light of recent advances in the understanding of bacterial secretion systems, the functional cooperation (i.e. several specialised secretion systems with a common goal) among them or their products is an interesting research area as it contributes to bacterial survival, rivalry, and virulence. However, the field of bacterial interaction barely comprehends this notion, given the lack of knowledge on the elaborate bacterial responses through multiple secretion pathways. As a result, the prevailing mind-set on multisecretion system-mediated bacterial response may currently be slanted among the research community. Secretion systems are well organized along a bacterial cell where they can operate either independently or interdependently of each other. Additionally, and as we later discuss, these secretion systems can also communicate remotely via the release of their functional molecules (Fig. 1).

Given that other reviews (e.g. [11–19]) have already covered the fundamental functions of secretion systems, we focused heavily in our review on the interconnections between bacterial secretion systems and how they impact bacterial biology. In order to further emphasize the importance of investigating bacterial secretion system interaction, we additionally offered a few previously underestimated functions that are mostly apparent during the interaction of the secretion systems, and provided their possible downstream implications.

## The general functions and biology of bacterial secretion systems

### TXSSs

With the exception of the T1SS, whose function is restricted to the dispersal of substrates into the external environment, the T3SS, T4SS, and T6SS of GNB are widespread double membrane spanning one step translocators of proteins into the environment or target cells without periplasmic passage (Fig. 1). The latter are contact-dependent injectosomes of GNB [20]. Recently, it has been shown that the mechanism for T1SS in some cases is a two-step process, and through bacteriocin-like protein surface aggregates, T1SS can facilitate antibacterial competition in a contact dependent manner [21–23]. This system typically secretes diffusible bacteriocins, adhesins, and proteins required during nutrient acquisition, such as iron scavenger proteins, lipases, proteases, and pore-forming toxins important for survival and pathogenesis [24, 25]. The T3SS and T6SS secrete effectors primarily involved in host immune subversion or manipulation and bacterial competition, respectively [22, 26–30]. Both these systems are also involved in other roles such as symbiotic interactions including mutualism, commensalism, and pathogenesis [4, 31–36]. As recently hypothesized, pathogenic bacteria may also use the T3SS to indirectly target microbiota populations [37]. On the other hand, additional roles of the T6SS have come to light in recent years, including anti-fungal activities and extracellular metal uptake [38–41]. The T6SS has recently been demonstrated to affect the competition between predator and prey cells inside the biofilm [41] and to transport a DNase that kills fungus by damaging their DNA via a Mg^2+^-dependent mechanism [42]. Unlike other TXSSs, excluding membrane vesicles (T0SS), the T4SS appears to have a unique function of delivering DNA and protein-DNA complexes, suggesting it is a tool for horizontal gene transfer (HGT) and, importantly, for spreading resistance genes among bacterial communities [43, 44]. However, the T4SS also shares some functions with other TXSSs, including bacterial killing and delivering effectors into host cells, functions previously thought to be unique to the T6SS and T3SS, respectively [45, 46]. Recent studies have revealed insights into the structure, function, regulation, and role of the T4SS in bacterial pathogenesis and host-pathogen interactions [47, 48]. The T2SS, T5SS, T8SS, T9SS, and T11SS are the GNB two-step transporters, among which, only the T2SS and T9SS act as transenvelope machines, spanning both the inner and outer membrane of the bacterial cell envelope [1, 49, 50]. Components of the T10SS that facilitate the release of substrates from the periplasm to the extracellular space have not been described [51]. The T2SS substrate repertoire continues to expand and includes typical substrates for bacterial adaptation and nutrient acquisition such as hydrolytic enzymes and toxins [2, 52]. Most substrates of the T5SS, and similarly of the T9SS, remain attached to the outer membrane and a few are released into the extracellular environment [7].

### Membrane vesicles

Bacterial membrane vesicles are arguably some of the most impressive features of microbes that have recently intrigued many scientists in the field of bacterial interactions. As vesicles exhibit some differences in transmission of molecular cargo compared to TXSSs, a brief discussion is warranted. Traditionally regarded as an inert membrane anomaly, membrane vesicles are frequently released by species in all three branches of life as subcellular lipid-bilayers (20–500 nm in diameter) [53]. According to a decades’ worth of research, membrane vesicles play a significant role in cell-cell and interorganismal communication due to their ability to internalize cellular contents (e.g. signalling molecules, toxins, proteins, metabolites, and nucleic acids) in their lumen. In this way, enclosed luminal contents maintain their potency because they are protected from extracellular degradative enzymes, making them highly concentrated and easy to deliver over long distances [54]. Upon release, membrane vesicles can either persist in the extracellular environment for long periods of time or fuse with prokaryotic or eukaryotic cells, for instance, via raft-dependent endocytosis, eliminating the need for microbes to have close contact with the host in order to rapidly transfer their cargo [55].

Bacterial membrane vesicles include outer membrane vesicles (OMVs), explosive outer membrane vesicles (EOMVs), cytoplasmic membrane vesicles (CMVs), and outer-inner membrane vesicles (OIMVs), mainly containing outer membrane (OM), cytoplasmic membrane, and outer and inner membrane (IM) components, respectively [56]. CMV, EOMV, and OIMV biogenesis happens through several proposed routes including explosive cell lysis via cryptic prophage endolysin activity [56]. OMV production entails unbalanced biosynthesis of cell wall components or the intercalation of hydrophobic molecules with the outer leaflet of the OM, leading to membrane curvature and eventual outgrowth of the OM [56–58]. For the purpose of this review, here on, all the vesicle types will be referred to as OMVs, for which formation is accompanied by the internalization of cellular contents delimited from extracellular enzymes degradation enclosed in the vesicle membrane [54].

## Intersecretion system-mediated response in bacteria

In this section, we explore some of the molecular mechanisms underlying the interconnectivity of the different secretion systems, and we show how this leads to several important functions, such as collaborative assault, setting-up intimate bacteria-host and bacteria-bacteria contact, exploitative competition, and horizontal gene transfer (HGT) in plant and animal bacterial pathogens as well as non-pathogenic bacteria. In Fig. 1 and Table 1, the interaction-mediated functions have been summarized, which, for the purpose of discussions in this section, are described in terms of substrate and system level interaction.

**Table 1.** Interactions among secretion systems in bacteria

Interacting system(s)*	Level of interaction†	Outcome and mechanism of interaction‡	Species	Ref(s)
T3SS-T5SS	Substrate level interaction (substrates: Tir and intimin)	**Cooperative**. Translocated Intimin Receptor (Tir) acts as a surface receptor for intimin, a T5SS substrate, owing to T3SS-mediated translocation of Tir.	Enteropathogenic *Escherichia coli *	[59]
T3SS-T4SS	Substrate level (BadA) and system level (T4SS) interaction	**Antagonistic**. BadA’s effective length creates a physical barrier between the pathogen and the host cell membrane, preventing T4SS activity.	* Bartonella henselae *	[65]
T2SS-T3SS	Substrate level (PCWDEs) and system level (T3SS) interaction	**Cooperative**. T2SS releases PCWDEs that alter plant cell wall integrity, thereby permitting T3SS-dependent effector protein translocation through.	* Xanthomonas citri* pv. *vesicatoria*	[74]
T1SS-T2SS	Substrate level interaction (substrates PrtA and PelI2)	**Cooperative**. T1SS substrate PrtA post-translationally modifies T2SS substrate PelI-2 to PelI-3	* Dickeya dadantii *	[73]
T4SS-T6SS	Substrate and system level interaction.	**Cooperative**. T4SS T4P promotes contact-dependent killing by T6SS by bringing adjacent prey closer to the attacker.	*Pseudomonas aeruginosa;* * Neisseria cinerea *	[86, 87]
T0SS-T6SS	Substrate level interaction (substrates: TseF and TeoL)	**Cooperative**. T6SS recruits T0SS (outer membrane vesicles or OMVs) either through its effector Tsef (* Cupriavidus necator *) or TeoL effector and PQS present in *Pseudomonas aeruginosa* OMVs.	*Pseudomonas aeruginosa; Cupriavidus necator*	[80, 81]
T0SS-T9SS	Substrate and system level interaction	**Cooperative**. T9SS effectors, typically extracellular cysteine proteases (gingipains), are anchored on outer membrane vesicles through anionic lipopolysaccharide (A-LPS) moiety of *Porphyromonas gingivalis* OM. This interaction results in a virulence coat contributing to *P. gingivalis* infections.	* Porphyromonas gingivalis *	[101]

*Type X secretion systems, where X stands for any number from 1 to 10, with outer membrane vesicles designated as a T0SS.

†Full names of molecules and components: BadA, *Bartonella* adhesin A; Tir, Translocated Intimin Receptor; TseF, T6SS effector for Fe uptake; TeoL, T6SS effector for recruitment of OMVs via lipopolysaccharide (LPS); PCWDEs, plant cell wall degrading enzymes; PQS, *Pseudomonas* quinolone signal; T4P, T4SS pilus. ‘Substrate’ refers to any molecule released by TXSSs (e.g. effectors, adhesins, etc.).

‡The term ‘outcome’ here refers to the way systems interact, which can be either synergistic or antagonistic.

### Host cell attachment and damage coupled to effector translocation

Contact with the host surface is perhaps one of the most important features of bacteria in initiating an infection, and it can trigger events in the host cell that promote a pathogen’s internalization and continuation of its life cycle [59, 60]. This is typically carried out by several T5SS classes, namely classical autotransporters (T5aSS), two-partner secretion (T5bSS), trimeric autotransporter adhesins (T5cSS), T5dSS, inverse autotransporters (T5eSS), and T5fSS [61]. Several of these T5SS classes reportedly function together with the T3SS and T4SS in establishing contact between the pathogen and the cognate adhesin receptor found on the host surface, and in some cases even driving cytoskeletal rearrangements and effector-triggered host cell invasion [62]. A classic example is the T3SS-dependent translocation of Tir (Translocated Intimin Receptor), a protein typically produced by enteropathogenic *Escherichia coli* (EPEC) bacteria; EPEC causes diarrhoea, the primary cause of morbidity and mortality among children in developing countries [63]. Tir plays a crucial role in the pathogenesis of EPEC infections and subsequent development of paediatric diarrhoea by promoting attachment of pathogenic *E. coli* cells to host intestinal cells and inducing changes in host cell behaviour. Following attachment, the T3SS of EPEC delivers a suit of effector proteins into the host cell, including Tir [59]. Tir becomes localized to the plasma membrane via the host Golgi apparatus [60], then acts as a receptor for a T5eSS substrate of the bacterial protein, intimin [59]. The established Tir–intimin linkage allows EPEC to form a tight adherence to the host cell, known as attaching and effacing (A/E) lesions, which emerge as a result of the effacing of the small intestine lining, a hallmark of EPEC infections.

The *Yersinia* adhesin A (YadA) from *Yersinia enterocolitica* and BadA (* Bartonella* adhesin A) from *Bartonella henselae* both fall under the T5eSS class. They engage in an intersecretion system-mediated translocation of effector molecules via the T3SS and T4SS [62, 64]. In particular, system interaction between BadA (T5cSS) and a T4SS core VirB/D-like subcomplex in *B. henselae* reduces T4SS mediated pathogen virulence [65], meaning the interaction is antagonistic unlike in EPEC [59]. Thus, most human *B. henselae* isolates were observed to have lost either BadA or VirB/D4 T4SS [65]. VirB/D4 denotes a structure of the T4SS that entails VirB1-11 proteins that assemble to form a secretion machinery and a pilus (T4P), and the VirD4 protein that is liable for substrate recruitment to the T4SS for secretion through the translocation channel [66]. The VirB/D4 T4SS function is thus interrupted by BadA which, through its effective length, enforces a physical distance (space) between the pathogen’s outer membrane (OM) and host cell membrane [65]. Subsequently, this negatively impacts the VirB/D4 T4SS effector translocation efficiency and ultimately impedes pathogen virulence. Considerations were made for possible shortfalls of the study resulting in the observed antagonism. It is possible that, in isolates where BadA and VirB/D4 co-exist to ensure persistence and effective infection, the strains likely express *BadA* or *VirB/D4* genes at different stages of host infection to, in part, minimise a speculated risk of cell wall instability due to protein overload in the OM. Also, given that most strains with either one of these virulence factors are observed, it is highly likely that they are antagonistic in these strains or the VirB/D4 is barely useful to retain in the presence of BadA. Such potential interactions have been missed in the literature as a result of isolated analysis of key virulence factors or the assumption that all secretion systems follow the same virulence factor-interaction strategies [65]. Observations relating to similar bacteria-host cell contact mechanisms that are mediated by secretion system interplay have been made for *Helicobacter pylori *, *Pseudomonas aeruginosa *, and *Salmonella enterica* [62, 67–72].

The T2SS and T3SS are identified as some of the key virulent determinants in bacteria and have been reported multiple times to promote host cell damage and virulence, respectively, in Gram-negative pathogens of plants and animals. It is not surprising that these two systems act in concert to achieve a bunch of objectives in bacteria. Specifically in plant infections, the T2SS of bacterial pathogens primarily export an arsenal of plant cell wall degrading enzymes (PCWDEs), such as pectate lyase, pectin lyase, polygalacturonase, cellulase, and protease [52]. In *Dickeya dadantii *, a broad-host-range enterobacterium belonging to plant pathogenic soft rot pathogens, there is an interplay between the T1SS and T2SS at the substrate level. During this interplay, T1SS extracellular protease PrtA post-translationally modifies the T2SS-dependent pectate lyase (PelI-2) by cleaving its *N*-terminal amino acids [73]. The resultant protein is a small, slightly more basic, and more efficient necrosis-inducing protein called PelI-3 [73]. PCWDEs such as PelI are delivered to the host cell surface whereby they act to reduce cell wall integrity, thereby establishing a pathogen’s nutrient supply line that is based on the cell wall components of the host plant. For this reason, it is conceivable that a portion of the cell wall-derived components will be utilized in the assembly of bacterial weapons including secretory channels of virulence factors, including the T3SS [26]. Here, we also demonstrate an interaction between this system and the T2SS in *Xanthomonas campestris* pv. *vesicatoria*, the causative agent of bacterial spot of pepper and tomato. The T2SS activity is thought to disrupt the plant cell wall by releasing hydrolytic enzymes that allow T3SS-dependent effector protein translocation [74]. The cell wall-degrading activity in *X. campestris* pv. *vesicatoria* is specifically carried out by the Xps-T2SS, one of the two T2SSs spanning the envelope of this pathogen [74]. Szczesny and co-workers [74] hypothesised that degradation of the plant cell wall helps in nutrient acquisition and that T2SS PCWDEs might facilitate the assembly of extracellular components of T3SS pili for effector injection into the host cell. The study highlighted that mutation of the Xps system reduced translocation of T3 effectors but did not markedly affect the T3SS or the synthesis of its components [74]. Traces of plant cell wall could be observed in some studies after it has been degraded. It is possible that PCWDEs weaken the plant cell wall for effective T3SS effector translocation. Further investigation is required to validated these postulated synergistic functions. Pili are often used for host penetration by bacteria since they can span the thick plant cell wall to translocate effectors into the target plant. Similar structures that are conserved in bacterial pathogens suggest that the T2SS shares composition and structural features with the T4SS pili (T4P) [75], important in bacterial adherence to host cells and other surfaces [76]. This enforces the notion of a common origin and potentiates pilus-mediated secretion, which is also involved in secretion system interaction (discussed later in this section). The interaction of the T2SS and T3SS is also tightly linked to the regulation of host defences, as well as the expression of genes and substrates of both systems, which is regulated by the HrpG/HrpX regulon [74, 77–79].

### A previously unrecognized iron acquisition and horizontal gene transfer mechanism

Direct physical contact of one bacterial cell with cells in the vicinity is an essential component of bacterial survival within a community. The T6SS is a bacteriophage-like machinery that is usually deployed during bacteria-bacteria contact where it delivers toxic effectors directly into neighbouring cells of competitor bacteria. All things considered, the T6SS is mostly regarded as a contact-dependent system, however, as we will show later, it can also engage in contactless exercises through interaction of its substrates with OMVs. Currently, there are no general mechanisms defining exactly how bacteria release, recognize, and recruit OMVs in an intra and inter-specific manner. However, two recent studies have revealed some key molecular mechanisms that might be involved in some of these processes in *P. aeruginosa* and *Cupriavidus necator *, entailing T6SS-mediated recruitment of OMVs during species communication [80, 81]. There are some aspects of this recruitment mechanism that are shared by both bacterial pathogens, and may also be found in other species. To begin, the T6SS effectors are employed as an OMV recruitment tool, and when OMVs are brought into play, a non-contact apparatus in the form of T6SS-OMV emerges [80, 81]. This makes reasonable sense as research shows that bacterial species can respond to environmental stimuli thanks to the release of OMVs [2]. Iron is an important metal for bacterial survival and virulence. *P. aeruginosa* H3-T6SS promoters in PAΔ3Fe (an iron acquisition mutant strain defective in the pyoverdin biosynthetic pathway (Δ*pvdA*), the pyochelin synthetase (Δ*pchE*) and the ferrous iron transport (Δ*feoB*) are induced in iron-deficiency conditions [81]. Under iron depleted environments, *P. aeruginosa* is able to scavenge iron from the extracellular 2-heptyl-3-hydroxy-4-quinolone (PQS), mostly found in OMVs. First, TseF, a H3-T6SS secreted effector, directly interacts with the iron acquisition receptor, FptA, then PQS, whose affinity with TseF increased in the presence of iron. Ultimately, TseF indirectly facilitates iron acquisition by delivering the OMV associated Fe^3+^-PQS complex iron to *P. aeruginosa* cells in a PQS dependent manner. More so, TseF bridging of the interaction between *P. aeruginosa* OMV-bound iron binding molecule PQS and *P. aeruginosa* cells by directly binding FptA [Fe(III)-pyochelin receptor] and porin OprF, consequently directs iron to the cells for uptake via an unknown mechanism. As expected, the growth of TseF and H3-T6SS mutants in iron depleted or Fe^3+^-PQS supplemented media, even in the presence of functional iron receptors, is severely affected in the absence of the recruiting effector, TseF [81].

The delivery of effectors or ions into target cells, even at a distance, from the site of colonization presents a unique population feeding advantage over traditional secretion systems that are often tightly fixed to the membrane and peptidoglycan layer of the cells. As noted previously, the T6SS primarily depends on contact to induce an effect on the recipient cells. Therefore, by recruiting OMVs through the incorporation of effectors into OMVs, the T6SS may perform its functions without proximity restrictions. Second, effectors that associate with OMVs for function or transport might also direct OMVs to the bacterial cell surface where they interact with specific OM receptors involved in iron uptake [80, 81]. Third, under iron-diluted conditions, activation of surface-associated receptors facilitates the delivery of iron to the cytosol, and once replete, dissolved iron can promote the competitive ability of *P. aeruginosa* and *C. necator* in their respective environments [80, 81]. When all of these factors are considered, a novel function of the T6SS, seen to promote the efficient utilization of iron when its sources are running low seems to be an intricate one.

We further consider the unique feature at play during the interaction of the T6SS with OMVs that is required for the bacteria to thrive in an iron-diluted environment. First, in *P. aeruginosa *, some aspect of iron uptake is facilitated by a newly described effector, called PA2374 or TseF, leading to the delivery of iron into *P. aeruginosa* cells [81]. In *C. necator *, a ligand (lipopolysaccharide)-receptor (CubA and CstR) interaction mediated inter-species OMV recruitment mechanism is observed. The LPS-binding effector TeoL [T6SS effector for recruitment of OMVs via lipopolysaccharide (LPS)] especially recognizes and binds the LPS on OMVs from different species (e.g. *C. necator* and distantly related *P. aeruginosa* PAO1 and *Yersinipseudotuberculosis* YPIII) containing the iron-chelating molecule PQS. Next, CubA (cupriabactin siderophore receptor) and CstR (catecholate siderophore receptor), both of which are part of the bacterial OM and essential for the ability of bacteria to grow and survive in iron-poor environments, are activated, eventually leading to ligand-receptor interaction-based OMV recruitment [80]. The *O*-antigen component of OMVs, which is a carbohydrate structural region of the LPS, and the compositional differences in LPS between bacterial cells and OMVs (e.g. partial loss of the LPS in OMVs), contribute to TeoL’s preferential binding to bacterial OMVs rather than to bacterial cells [80]. In addition to iron uptake, the OMVs recruited by TeoL were observed to be important for exploitative competition, resistance to oxidative stress, and HGT in recipient cells—key in their survival and persistence. Given the fact that PQS and LPS are involved in OMV production during a process entailing PQS-mediated anionic repulsions between the LPS molecules [82], it would be interesting to examine the possibility of effector-induced vesiculation in bacteria as a way to increase nutrient sources that are depleted. This brings up the issue of whether OMVs could be utilized as a wellspring of nutrients or factors significant for nutrient acquisition, and whether TXSS effector-mediated recruitment of OMVs is a general survival mechanism used by bacteria. Nonetheless, the discoveries of these studies certainly illuminate how we might interpret the role of bacteria in recognizing and recruiting OMVs for various survival and host invasion strategies.

T6SS-mediated recruitment of OMVs also enables bacteria to participate in HGT. OMVs can drive HGT and bacterial resistance to stress [83–85], but to our knowledge, they have seldom been associated with other TXSSs in accomplishing HGT. Recently, it was established that the T6SS can promote HGT by enabling acquisition of DNA from OMVs purified from bacterial cultures containing plasmid DNA [80]. As previously discussed, pilus-mediated interaction in terms of intersecretion system crosstalk plays a key role in bacterial contact with either other bacteria or the host. A key example of this is a pilus-mediated interaction in terms of HGT that involves a crosstalk between the T4SS and T6SS (Fig. 1) [86]. This is consistent with HGT encompassing T4SS-mediated cell-cell contact through conjugative DNA transfer in bacteria [76]. The T4SS-mediated HGT was found to activate T6SS-mediated killing of adjacent donor cells carrying parasitic foreign DNA, and is considered an ‘innate immune system’ that recognizes transfer-associated patterns instead of molecular patterns of infectious elements [86]. When contact-dependent killing via intersecretion system crosstalk is involved, this suggests that HGT is undeniably more complex than previously thought.

### Intersecretion system-mediated microbe-microbe contact

The interaction between the T6SS and T4SS has recently been observed to play a role during contact between non-pathogenic *Neisseria cinerea* and other human pathogens [87]. As mentioned previously, T4Ps form part of the VirB/D4 T4SS design, and are found on bacteria’s surfaces, aiding in bringing bacteria into close physical association with host cells and other bacteria [88]. In competition assays involving human commensal and pathogenic strains of *Neisseria *, *N. cinerea* was instrumental in killing pathogens, *N. meningitidis* and *N. gonorrhoeae *, in a T6SS-dependent manner [87]. Prey strains of *Neisseria* lacking a T4P, in particular, were able to escape the T6SS-mediated killing by segregating to the edge of the colony seeded on agar medium. However, prey strains that expressed a pilus were outcompeted by the killer strain due to cellular interaction between themselves and *N. cinerea *, which was mediated by a T4P. This implies that T4P promotes the activity of contact-dependent TXSSs by bringing prey closer to the attacker. It is not uncommon for bacteria to influence the outcome of an infection by directly killing other bacteria through the antibacterial action of the T6SS. This has been associated with changes in microbial communities and a range of ecological consequences [89]. Therefore, intersecretion system as a function of pilus-mediated interaction may greatly contribute to microbial community structures and composition, which is reminiscent with the role of the T6SS [89].

### Shared TXSS substrates highlight a conceivable interplay

Although there are a number of studies which do not directly dissect bacterial secretion system dialogue, they give significant insights to their possible interaction and set the stage for future research. For example, the *P. aeruginosa* T3SS and T6SS may work together to regulate transcription factors that activate unique transcriptome changes during early airway epithelial cell infection [90]. With respect to the T3SS and T4SS, we speculate functional interplay could be a possible outcome since the effectors of these secretion systems are often observed to be remarkably alike in structure and function [91]. This means that the functional secretion system might act in place of the mutant system and secrete its substrates. Alternatively, the functional secretion system substrates might substitute the mutated systems’ substrates. In addition, several studies on bacterial secretion suggest that many substrates are shared between OMVs and TXSSs. For instance, OMVs are known to export many proteins which play a role in bacterial virulence and communication, and many of these are shared with TXSSs [54]. In plant-associated bacteria, a large number of studies reported diverse proteins in OMVs that are biologically important [92]. An overlap in substrates between the T2SS and OMVs was observed, whereby *X. campestris* pv. *vesicatoria* strains lacking a functional T2SS independently secreted several substrates of the T2SS system, including extracellular protein cargoes such as lipases, proteases, and cell wall–modifying enzymes via OMVs [93]. Likewise, OMVs isolated from phytopathogens overlap in extracellular protein cargo with the T2SS and T3SS [94, 95]. Both T2SS secreted hydrolytic enzymes and the T3SS effectors whose translocation into host cells is potentially facilitated by them were found in OMVs of *P *. *syringae* pv. tomato T1 [94]. In addition, OMV-mediated transport of biologically active T2SS dependent PCWDEs has been reported in *Pectobacterium* spp. *P *. *brasiliense *, *P. odoriferum, P. versatile* and *P. zantedeschiae* and *X. fastidiosa* vesicles [96–98]. Taken together, these analyses suggest that OMVs could serve as an alternative secretory pathway for other TXSSs in bacteria or act in coordination with them.

## Implications for the inter-secretion system dialogue

Interaction between secretion systems could be a common occurrence in prokaryotic organisms. How it impacts bacterial function and interactions is not yet clear, but it perhaps represents an ingenious mechanism that ensures bacteria use adequate tools at the right time to enhance their fitness potential. In this section we consider some of these specific aspects and their potential consequences.

### Membrane vesicles shared as ‘public goods’

Although OMVs were originally thought to be merely membrane artefacts with no clear cellular importance, in recent decades, an enormous number of investigations gave an account of their functions including in nutrient acquisition and exploitative competition. For example, OMVs from *Mycobacterium tuberculosis* can carry high amounts of an iron chelating molecule, myobactin, which forms iron-scavenging OMVs [99]. Once released into the environment, these myobactin-OMVs can be shared *bona publica* as they will be relatively easy to access by neighbour bacteria as a community resource, thereby contributing to the social life of that community [100]. In essence, OMVs can serve an ecologically significant role for the successful coexistence of different bacterial species in the same habitat or within biofilm–structured communities of microbial aggregates enclosed in a self-produced polymeric matrix and attached to biological and non-biological surfaces. Here, we consider these ideas in the context of OMV interaction, which so far have been reported with the T6SS and T9SS. As previously noted, *P. aeruginosa* secretes OMVs carrying on their surface the PQS molecule, which, like myobactin, also strongly binds iron. These PQS-containing OMVs can be recruited by the T6SS effector TseF [81], and possibly by other members in the bacterial community that express the T6SS effector. Although further research is needed to determine the entire range of bacteria that secrete this effector, it is possible that other bacteria may also produce the effector. Additional proof of OMVs as potential public goods relates to features of the bacterial OM. The LPS, which is one of the major constituents of bacterial cells and OMVs, is important for the cell envelope of GNB, serving principally as a structural component of OM and released OMVs. Likewise, there is solid support of OMV-mediated conveyance of the LPS among bacterial species which can also trigger important host cellular processes [101–103]. This solidly addresses the ability of bacterial cells to disperse significant surface-confined particles even to locales where OMV donor cells themselves cannot reach. Along these lines, the T6SS-mediated recruitment of OMVs may hypothetically be an element of bacteria traded among neighbours and as a public good. Considering that the T6SSs are present in a wide spectrum of Gram-negatives (>25 % of Proteobacteria) [104], the T6SS-mediated recruitment of OMVs could therefore be an alternative mechanism of utilizing scarce iron from its dilute sources exploited by many bacteria. In addition, iron acquisition in this manner also brings about other benefits, as we have seen that the capacity of the T6SS effectors to recruit OMVs also prompts other important functions, including HGT and stress tolerance, that are critical for bacterial rivalry and survival while sharing the same habitat [80, 81, 105]. In a similar scenario, the T9SS, a translocon in a few *Bacteroidetes* spp. including *Porphyromonas gingivalis *, interacts with OMVs via a battery of virulence effectors (Fig. 1) [101]. These include extracellular cysteine proteases, representing hallmark virulence factors of this pathogen, commonly called gingipains (RgpA, RgpB, and Kgp) [101]. These virulence factors carry a conserved C-terminal domain (CTD) that is used as an extracellular OM translocation signal by the T9SS [106]. Following translocation, the CTD is cleaved off by a bifunctional C-terminal signal peptidase and sortase enzyme, PorU. The sortase is also released via the T9SS but, unlike gingipains, it retains the CTD signal [106–108], after which its replacement, as well as that of gingapins, with a unique anionic lipopolysaccharide (A-LPS) moiety of *P. gingivalis* OM effectively anchors the released proteins to the OM, forming a virulence coat on the cells and OMVs [101, 108, 109]. Because the LPS can be exchanged between bacterial strains, the OMVs coated with virulence factors (virulence factor-coated) may be released into the extracellular space, where they will most likely be shared as public goods [101, 103]. In this way, the virulence coat can be spread by released OMVs, and in some cases even to other non-pathogenic *P. gingivalis* strains within the pathogen population. Noteworthy, bacteria can trade certain traits through the use of OMVs [103]. Such a vesicle-mediated exchange of cargo or traits is not confined to bacteria. In the yeast genus *Cryptococcus*, for instance, extracellular vesicles released by virulent strains can trigger a rapid intracellular proliferation of non-virulent yeast strains residing within the macrophages [110]. This results in pathogenic ‘division of labour’, which occurs remotely where vesicles diffuse over large distances, in the process possibly transmitting virulence factors to these strains. Alternatively, it is possible that the *Cryptococcus* vesicles can modify the host environment to allow less pathogenic strains to thrive, which would also facilitate the pathogenic ‘division of labour’. The same ‘division of labour’ phenomenon may apply in *P. gingivalis* strains when the T9SS virulence coat is distributed from a few pathogenic members to otherwise latent and non-virulent strains.

Conjugation is a widespread channel of HGT, a consequence of which is rapid evolution and adaptation of bacterial strains through the spread of antimicrobial resistance genes [111]. Plant-derived bioactive compounds identified to inhibit T4SS-mediated conjugal transfer of plasmids without perturbing GNB growth were reported not long ago [112]. However, in addition to conjugation and transformation by the T4SS, OMVs are also disseminators of antibiotic resistance genes in GNB, and cultures supplemented with purified vesicles are protected from antimicrobial compounds by several strategies such as drug binding [113–116]. In *Vibrio cholerae *, a protease essential in bacterial resistance to host antimicrobial peptides and conventionally secreted via the T2SS, is associated with OMVs [117]. Being evolutionarily conserved, constitutive, and primarily produced in response to stress, dissemination of resistance via OMVs may soon become notably problematic as resistance genes continue to be availed to complex microbial communities [118]. We are likely to observe roles beyond OMV-T2SS, -T3SS, -T4SS, -T6SS, and -T9SS associations as vesicles are handy and convenient sources (i.e. public goods) of protected and concentrated bioactive compounds for mixed communities [3, 119].

### Differential secretion of substrates

The focus of profiling secretion systems includes proteomic research. To demonstrate secretomes that are representative of the function of the missing system, secretion system knockout mutants are often constructed. In proteomic investigations, it has been revealed that proteins are downregulated in TXSS mutant strains compared to wild-type strains in the hunt for the TXSSs' major substrates. It is possible that the differentially secreted proteins are an indication of secondary protein secretion or regulation by the disrupted system. The key to bacterial fitness and survival in the environment is synchrony. Bacteria intentionally stimulate protein secretion for this reason so they can react quickly to environmental changes [120]. In this way, regulatory networks, and secretion systems along with their crosstalk play a critical part in the communication, which is in turn critical for fitness in all forms of life [121]. For instance, a recent study identified new *D. dadantii* T2SS substrates displaying a band of low intensity and thickness for one protein, VirK, in the supernatant of the T2SS inactive strain as compared to the supernatant of the T2SS active strain or the complement [122]. VirK could have leaked, or in our judgement was a subject of secondary secretion by OMVs or another system thus the cause of its appearance in T2SS mutant secretomes. Similarly VirK and a cellulase were previously only discovered in the minimum media with extract of the T3SS active strain secretome of the phytopathogen *X. citri* subsp *. citri* [123]. The study attributed the outcome to the dysfunctional T3SS system in the mutant strain or that the T3SS apparatus had pleiotropic effects on the expression and secretion of some proteins. However, since another study demonstrated in *Ralstonia solanacearum* that HrpG of the T3SS regulated VirK, it was concluded that the T3SS controls T2SS secretion [123]. A study in *Salmonella* identified the crosstalk between the T6SS encoding SPI-19 and the T3SS in avian infection, which is another recent discovery [124]. The research revealed that T3SS regulators made up part of the T6SS island. Genes that make up the T6SS core components were not a part of the observed regulation [124]. All things considered, regulatory or polar effects of some mutations may be at play. Nonetheless, it is crucial to keep in mind that secretion systems could be involved in co-secretion and species dependent secretion of substrates in addition to substrate and systems level interaction. The most recent computational resource for identifying novel substrates is called BastionX. It has predictors for the secretion systems T1SS-T4SS and T6SS and calculates probabilities of secreting a given protein by each system. Some proteins seem to fit profiles with the best possible score using this method as substrates of at least one secretion system [125].

Bacteria must have mechanisms in place to carefully control secretion in order to produce smooth and effective interaction mechanisms, and we expect to be able to properly respond in case a system fails. In this review, the effects of secretion systems crosstalk have been emphasized to show potential consequences of both harmonious and antagonistic action. It is obvious that specific TXSSs' activities have an impact on how well other secretion systems function. One potential effect of a dialogue is compensatory behaviour among secretory systems to attain collective goals in light of the impacts. It is a frequent misconception that a secretion system only carries one protein [1]. Bacteria likely modify these underutilized systems to transport additional substrates with a similar structure to their principal substrates. Therefore, it is essential to develop methods to study the full-circle circuitry of protein secretion. Outputs will reveal data required to understand the complexities behind secretion system overlap and implications in crucial areas like the use of secretion system inhibitory strategies for disease control.

### Prediction and screening of secretion system substrates

Secreted proteins and their functions delineate what a secretion system is used for [7]. A portion of secretomes typically studied *in vitro* includes effectors that facilitate interactions between producing bacteria and their hosts. Bioinformatics, proteomics, and biochemical approaches are used to study secretomes. In addition, databases such as Bacterial Secreted Effector Protein DataBase (SecretEPDB) harbour pre-calculated knowledge bases of reference effectors [91]. Computational pipelines use the available secretion systems substrate data (sequence features, e.g. secretion signals and conserved motifs) to predict candidate effectors from available microbiota genomes and proteomes. To date, over 40 bioinformatics tools are available to predict GNB T1SS-T9SS substrates [126]. Bioinformatic tools often rely on two strategies: sequence similarity to known effectors and identification and analysis of unique gene islands of a pathogen. However, there is a limited number of experimentally validated secretion systems cargo, and the poor specificity of prediction tools poses significant challenges to the similarity and unique island predictions' efficiency [126, 127]. Poor specificity is partly due to the construction of some algorithms and tools for specific bacteria or substrates [126]. Another limitation of *in silico* screening of effectors is that most predictors are designed to screen proteomes for substrates of a single secretion system, further limited by a bias towards specific effector groups. Hui and co-workers [126] presented 45 representative single secretion system substrate predictors. This group is populated with machine learning algorithms based T3SS and T4SS predictors. Recent advances seek to delimit computational prediction limitations by constructing tools that predict effectors for several secretion systems. One recent semi-knowledge based multiple secretion systems substrate predictor is PREFFECTOR [128]. The most recent computational resource for screening for novel TXSS secreted substrates is BastionX [125]. It has substrate predictors for the secretion systems T1SS-T4SS and T6SS and calculates probabilities of each system secreting a given protein. The upside of this tool is that it predicts both homologs of experimentally validated effectors and novel distant candidates [125]. Computational pipelines have been utilized to some degree to predict host-pathogen-effector triggered susceptibility agents and pathogen associated molecular patterns or effector triggered immunity protein interactions [129]. Developing predictors with broad prediction capabilities will go a long way in screening GNB proteomes for candidate effectors. Bioinformatic approaches used alongside approaches such as proteomics and phage display technologies take the screening process for candidate effectors a step further. Mass spectrometry coupled with bioinformatics is used to identify and annotate proteins in secreted protein sample preparations, thus further filtering the *in silico* predicted candidates and identifying secretomes unbiasedly. Phage display affinity screening identifies proteins implicated in interactions of microbes with their environments or hosts through virion facilitated physical interactions with target proteins [130]. In our view, these platforms offer a means to identify, functionally annotate proteins, and establish protein-protein interaction networks between self (in this case, secreted effectors of multiple systems) and environmental or host proteins. The networks might give insight into probable functions and functional links of proteins secreted by different systems that would guide downstream undertaking applications, i.e. disease management. To study substrate cooperation or co-secretion by OMVs, T1SS-T4SS, and T6SS, reporter-based assays can be used. Guided by the secretion systems under inspection, substrates would be cloned as fusions with detectable tags that do not impact secretion (i.e. neutral tags) and expressed by transformed strains for selective identification using affinity chromatography, fluorescence microscopy, enzymatic assays, or Western blot from the wild-type and single or multiple secretion system inactive strain secretomes. Tags associated with an enzymatic activity provide quantitative data on secretion. Densitometry can be used to quantify relative band intensities [131]. The final step would be to seek experimental evidence to support the proposed biological roles of the secreted proteins as effectors.

## Conclusion

We have emphasized that secretion systems can be interconnected and, in some cases, interdependent through physical contact or by delivering molecular payloads – like effectors and other key proteins into the environment or target cells. By retaining such an elaborate capacity to enable interactive system communication in their cells partly helps us explain why bacteria can thrive in their natural habitats through a number of functions; for one, bacteria, among many roles, could derive an agile response to cope with a dynamically changing environment. In other words, this phenomenon could be behind some of the complex phenotypes commonly encountered when multigene pathways are involved, such as bacterial resilience to stress and antimicrobial agents. Be that as it may, this review in no way implies that any of the discussed interactions or their effects serve as a universal mechanism for bacteria to interact with one another or with their hosts. The review only seeks to highlight the magnitude of any beneficial or harmful effects resulting from interactions amongst secretion systems and the potential consequences as these interactions evolve. The absence of empirical data for a crosslink between these secretion systems represents a significant barrier to analyse their effects in bacteria. Therefore, it is envisioned that taking advantage of the complexity of these linked operations holds out the prospect of new horizons to be explored, such as new treatment strategies for complex bacterial diseases in animals or new plant improvement prospects.

## New direction

Considerable efforts are necessary to unpack the molecular mechanisms underlining the convergent function of secretion systems, which may open up new possibilities. As this area is one of a few enigmas in bacterial interactions, research behind the system interaction is likely to gain traction, and a broader consequences of cooperative or antagonistic behaviour that is primarily directed at maintaining adaptability in bacterial responses in the natural environment would emerge. Such details could present an attractive model for developing broad spectrum or multisecretion system antivirulence compounds to alleviate the negative consequences of bacteria, or simply to study the well-coordinated phenotypic developments in prokaryotic species.
